# A Comparison of Statistical Analysis Between "Real" Patients Reported in Kaplan-Meier Curves and "Reconstructed" Patients Estimated Through the IPDfromKM Method: Analysis of Eight Trials Evaluating Catheter Ablation of Ventricular Tachycardia

**DOI:** 10.7759/cureus.47891

**Published:** 2023-10-29

**Authors:** Andrea Messori, Valeria Fadda, Maria R Romeo, Sara Veneziano, Sabrina Trippoli

**Affiliations:** 1 Health Technology Assessment (HTA) Unit, Tuscany Regional Health Care System, Firenze, ITA; 2 Pharmacology and Therapeutics, Ente di Supporto Tecnico Amministrativo Regionale (ESTAR), Firenze, ITA; 3 Health Policy, Fondazione Toscana Gabriele Monasterio, Massa, ITA; 4 Pharmacology, University of Pisa, Pisa, ITA

**Keywords:** relative risk, hazard ratio, meta-analysis, indirect comparisons, time-to-event endpoint, kaplan-meier statistics

## Abstract

Time-to-event endpoints are most widely used in oncology and, to a lesser extent, in cardiology. Typical statistical parameters employed in this context include overall survival, progression-free survival, and recurrence-free survival. The graphical presentation of the results is based on the Kaplan-Meier plot. When Kaplan-Meier curves are included in a meta-analysis, the typical methodological approach is a simplified one because the results of each trial (as well as those of the meta-analysis itself) are expressed through a 2x2 contingency; the methodological simplification is that the follow-up is left out from the analysis and, consequently, the Kaplan-Meier curves are omitted as well. The IPDfromKM method, developed in 2021, is an artificial intelligence algorithm designed to be used in these situations. According to this method, to keep the Kaplan-Meier curves in the meta-analysis, each curve is converted into a database of individual patients (which are denoted as "reconstructed" individual patients). In this way, for the purposes of the meta-analysis, the statistical methods are based on individual patients (like those of clinical trials) so that the Kaplan-Meier curves must not be excluded, and the effect of the follow-up can, therefore, be investigated.

This technical report describes the IPDfromKM method in all of its operational details. To present the method, a meta-analysis investigating the effects of catheter ablation to prevent ventricular tachyarrhythmia (VT) has been taken as an example. The original meta-analysis, which included nine controlled trials, was published in February 2023 and adopted the simplified approach based on 2x2 contingency tables. We have reanalyzed these trials by using the IPDfromKM method. Overall, both the standard binary meta-analysis and the IPDfromKM method showed that ablation significantly reduces VT recurrence (hazard ratio, 0.820 for binary meta-analysis vs 0.728 for the IPDfromKM method). By contrast, while no heterogeneity was found by the binary method, the IPDfromKM found significant heterogeneity, which was confirmed by visual inspection of the Kaplan-Meier curves. This suggests that the results of the IPDfromKM method are more accurate because they include the effect of the follow-up on patients' outcomes. In conclusion, our reanalysis confirms the significant benefit determined by ablation, but a more pronounced degree of between-trial heterogeneity has been found. Finally, it should be stressed that, outside the field of meta-analysis, the IPDfromKM method is also applicable to carry out an indirect comparison between treatments that have never been compared in real clinical trials. In this case, reconstructed patients are analyzed by conducting a simulated comparative trial.

## Introduction

When a clinical trial is based on a time-to-event endpoint, its final results are generally presented through a Kaplan-Meier plot. Time-to-event endpoints are most widely used in oncology, and, in fact, overall survival (OS), progression-free survival (PFS), and recurrence-free survival (RFS) have been considered for many decades a gold standard in the field of endpoints. More recently, time-to-event endpoints are also increasingly being used in cardiology, where they often have the nature of composite endpoints.

The presentation of trials' results includes a Kaplan-Meier curve, especially when there is a long follow-up and, at the same time, the clinical impact of the follow-up is important. In contrast, when the follow-up is unimportant, the results can be presented in simplified form (often denoted as meta-analysis of "binary" outcomes or binary meta-analysis); this implies reliance on a simple 2x2 contingency table. In managing the risk of the event, the typical statistical parameter is the hazard ratio in the case of a Kaplan-Meier plot; by contrast, when a 2x2 contingency table is used, the typical statistical parameter is the relative risk or the odds ratio.

Meta-analysis and indirect comparisons are two areas of evidence-based medicine in which time-to-event endpoints and Kaplan-Meier curves offer an improved interpretation of the results, particularly when the endpoint depends on the follow-up. Of course, this holds true, particularly when the follow-up has a duration of many years. In both these areas, the statistical parameters recommended by current methodological standards consist of 2x2 contingency tables, thus representing a very simplified methodological approach. Until recently, the statistics employed in the two above-mentioned areas of evidence-based medicine did not analyze Kaplan-Meier plots owing to the lack of well-recognized statistical procedures and related ad hoc statistical software. Consequently, the great majority of the scientific reports published in these two areas have employed a simplified design, i.e., have employed 2x2 contingency tables. This limitation has determined a lack of analyses in which the effect of the follow-up on these endpoints has been investigated.

At the beginning of 2021, this scenario has changed because new methods for managing the information reported in Kaplan-Meier curves have been developed. The most important one is the IPDfromKM technique [1], published in June 2021, which is a method that employs, at the same time, statistical algorithms and artificial intelligence. The IPDfromKM method has a unique characteristic because the analysis of each Kaplan-Meier curve is aimed at generating the corresponding archive of the so-called "reconstructed" patients. Hence, the researcher submits the Kaplan-Meier plot to the computer program (along with some basic information on the clinical trial concerned), and the computer program delivers to the researcher a detailed archive of "reconstructed" patients. In this way, the researcher can include these reconstructed patients in any type of multiple-trial analysis or in any head-to-head simulated comparative trial (which can evaluate treatments that have never been compared in the "real" world). Interestingly, a wide body of data has demonstrated that the quality of the reconstruction operated by the IPDfromKM software is excellent. In these reports, the IPDfromKM approach has been denoted as the method of reconstructing individual patient data and also as the Shiny method.

Between 2021 and 2023, numerous studies have used this new approach based on reconstructed individual patient data. Most of these investigations fall in the areas of oncology [2,3] and cardiology [4]. Finally, the present technical report differs from a similar article published in 2021 [5] because a more detailed example of the application of the IPDfromKM method is presented herein.

## Technical report

In this technical report, the standard binary meta-analysis published in 2023 by Virk and Kumar [6] is taken as an example. Briefly, this meta-analysis examined nine controlled trials conducted in patients at risk for ventricular arrhythmia and compared patients treated with catheter ablation of ventricular arrhythmia with a group of controls not receiving such ablation. The main endpoint was the recurrence of ventricular tachycardia (VT) over time. In these nine trials, this endpoint was managed by Virk and Kumar by constructing a 2x2 contingency table with no adjustment for the follow-up length. In our reanalysis of the same data, we instead employed the IPDfromKM approach [2].

In the present section, we will first re-examine the results reported in the meta-analysis by Virk and Kumar [6]. Then, these results will be compared with those obtained through the analysis of the same data, performed by application of the IPDfromKM method.

Standard binary meta-analysis published by Virk and Kumar 

The characteristics of the nine clinical trials included in the meta-analysis by Virk and Kumar [6] are summarized in Table 1. The main result of the meta-analysis is the following: compared to the control groups, catheter ablation significantly reduced VT recurrence; the relative risk (RR) was 0.82; I2=0%; 95% confidence interval (CI), 0.70 to 0.97; p=0.02.

**Table 1:** Characteristics of the nine RCTs included in the meta-analysis by Virk and Kuma § This column indicates the specific endpoint employed in each trial to construct the Kaplan-Meier curve published in the original article. §§ In the original report [6], the number of events observed in each trial (i.e., the numerators in columns 4 and 5) were not reported analytically; this information as presented in these two columns of this table refers to the archives of reconstructed patients generated by application of the IPDfromKM method. n - number of events; N - number of patients; VT - ventricular tachycardia; VF - ventricular fibrillation; ICD - implantable cardioverter defibrillator; AAD - antiarrhythmic drugs.

First author	Trial acronym	Endpoint definition^§^	Control arm	Outcomes^§§^ (n/N)		Notes	Trial identification code
				Ablation arm	Controls		
Arenal, 2022 [7]	SURVIVE-VT	Appropriate ICD shock	AAD therapy	12/71	13/73		1
Della Bella, 2022 [8]	PARTITA	VT recurrence with shock	Medical therapy	2/23	6/24		2
Kuck, 2010 [9]	VTACH	VT or VF recurrence	ICD+medical therapy	27/52	39/55		3
Kuck, 2017 [10]	SMS	Time to first VT/VF recurrence	ICD implantation without catheter ablation	20/45	24/60		4
Reddy, 2007 [11]	SMSH-medical therapy	Survival free from ICD	Medical therapy	8/64	21/64		5
Sapp, 2016 [12]	VANISH	Survival free from VT recurrence	Escalation of AAD therapy	50/132	54/127	Survival free from ICD shock	6
Tung, 2022 [13]	PAUSE-SCD	Survival free from VT recurrence	ICD+medical therapy	19/60	31/61		7
Willems, 2020 [14]	BERLIN- VT	Survival free from ICD therapy	Medical therapy until the third ICD shock (then VT ablation)	13/73	18/83		8
Al-Khatib, 2015 [15]	CALYPSO	Endpoint of VT recurrence	Anti-arrhythmic drug therapy	8/13	6/14	Owing to the lack of a Kaplan-Meier plot, this study was not included in our re-analysis based on the IPDfromKM method	-

Re-analysis of the same trials performed by application of the IPDfromKM method

In examining the nine RCTs [7-15] studied by Virk and Kumar [6], we noticed that the CALYPSO trial [15] did not report any Kaplan-Meier graph. Consequently, our re-analysis included only 8 RCTs [7-14]. The results reported in the excluded trial (event rate of 6/14 in the ablation group vs 8/13 in the controls; RR, 0.70; 95%CI, 0.33 to 1.46; p=0.34) were based on an extremely small sample size. 

To run the IPDfromKM algorithm, the information about these trials that we entered into the software consisted of 8 Kaplan-Meier plots (one for each trial), with each plot containing two curves (one for the experimental group and the other for the controls). Besides these plots, the information uploaded in the IPDfromKM software included the numerical values reported in columns 5 and 6 of Table 1. It should be stressed that each plot of a Kaplan-Meier curve is extremely rich of important information about survival (including the number and the timing of events, the number and the timing of terminations of follow-up, information on the follow-up length, number of at-risk patients stratified over time, etc.).

In running the IPDfromKM algorithm, the Kaplan-Meier curves must be analyzed one at a time. The results of each of these analyses are represented by an Excel file containing as many rows as the number of patients. Overall, we therefore analyzed 16 curves reported in the eight included trials. Therefore, the results consisted of 16 Excel files containing the data of all the reconstructed patients. We analyzed this clinical material using standard survival statistics (Cox regression analysis). For this purpose, we used four packages ("survival", "survminer", "survRM2", and "readxl") that were run under the R-platform (R Foundation, Vienna, Austria) [16]. Regarding the results of our analysis, on the one hand, we generated 16 new Kaplan-Meier plots based on the reconstructed patients; on the other, we performed a number of survival statistics to estimate the hazard ratio (HR) for the comparison of experimental groups versus controls and to evaluate the heterogeneity across the included trials. Further methodological information on this issue can be found in References [2] and [3]. Finally, confidence intervals (CIs) were estimated where appropriate.

**Figure 1:**
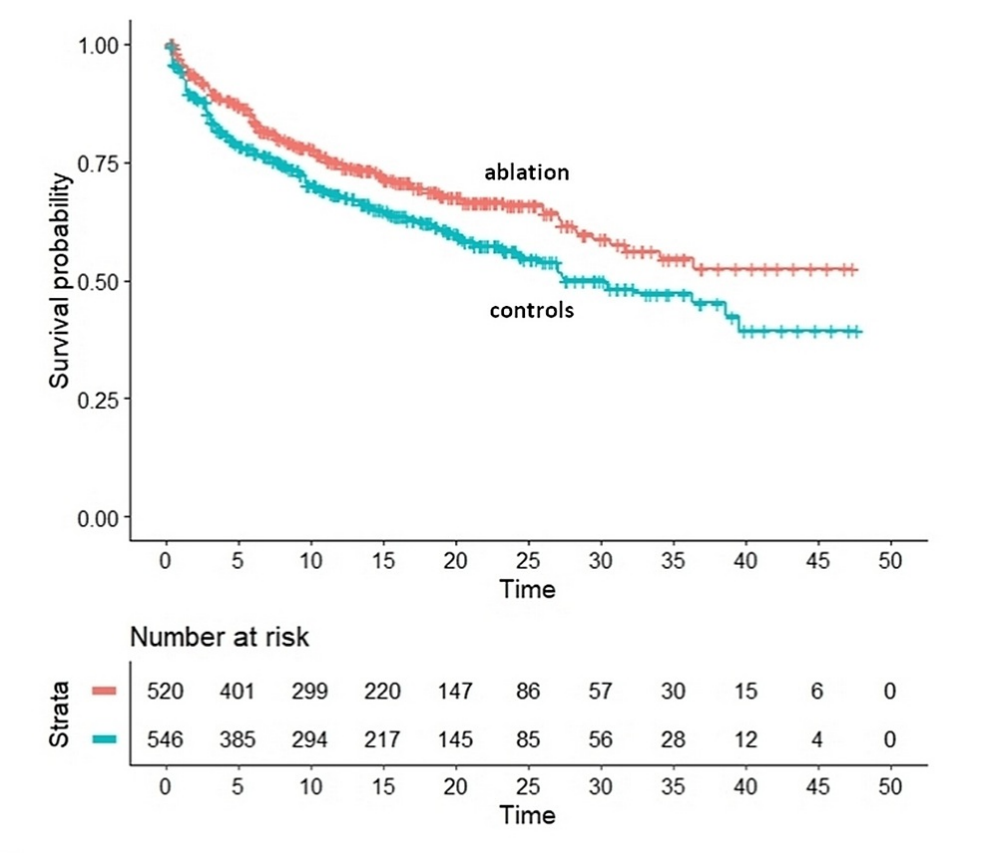
Graphical results of the analysis based on reconstructed patients (IPDfromKM method) Endpoint, recurrence of VT/VF or ICD shock; the Kaplan-Meier plots have been generated from reconstructed individual patient data; time is reported in months VT - ventricular tachyarrhythmia, VF - ventricular fibrillation, ICD - implantable cardioverter defibrillator

Figure 1 shows the graphical results of our analysis based on reconstructed patients. The outcomes were significantly better in the treatment group compared to the controls. Regarding this comparison, the HR was estimated as 0.728 (95%CI, 0.590 to 0.898; p=0.003). Median event-free survival was 31.8 months for the ablation group vs 24.7 months for the controls.

**Figure 2:**
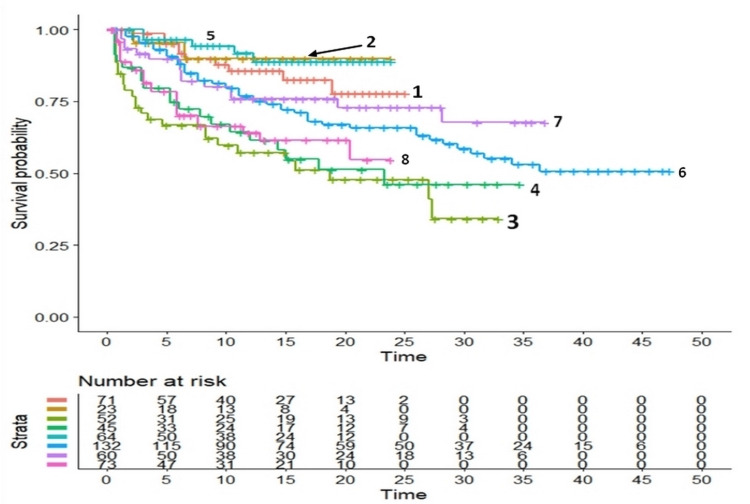
Graphical representation of between-trial heterogeneity in the eight patient groups treated with ablation Endpoint, recurrence of VT/VF or ICD shock; the Kaplan-Meier plots have been generated from reconstructed individual patient data Notes: a) the reference number denotes each clinical study according to trial identification codes reported in Table 1; b) in all Kaplan-Meier plots, time is reported in months VT - ventricular tachyarrhythmia, VF - ventricular fibrillation, ICD - implantable cardioverter defibrillator

**Figure 3:**
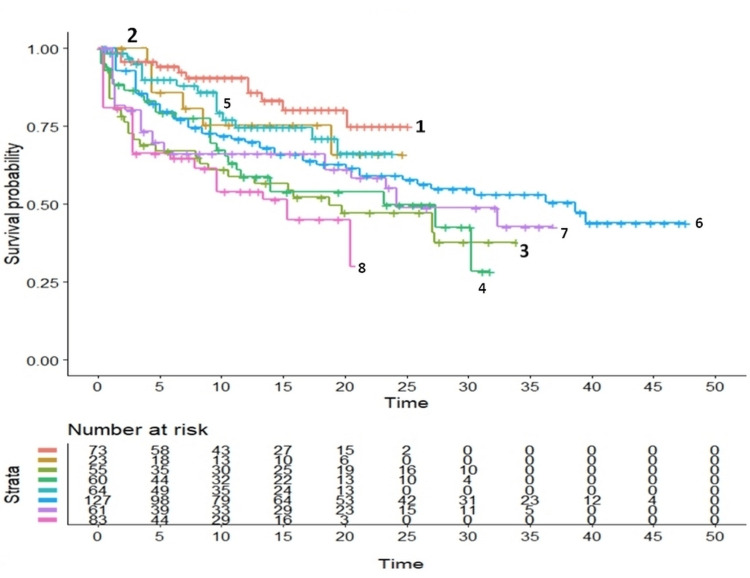
Graphical representation of between-trial heterogeneity in the eight control groups § Endpoint, recurrence of VT/VF or ICD shock; the Kaplan-Meier plots have been generated from reconstructed individual patient data Notes: a) the reference number denotes each clinical study according to trial identification codes reported in Table 1; b) in all Kaplan-Meier plots, time is reported in months VT - ventricular tachyarrhythmia, VF - ventricular fibrillation, ICD - implantable cardioverter defibrillator

Regarding heterogeneity, the graphical results for the ablation group and the controls are presented in Figure 2 and Figure 3, respectively.

These findings show marked between-trial differences both in the ablation group (Figure 2) and in the controls (Figure 3). In numerical terms, the assessment of heterogeneity estimated from the eight curves of patients subjected to ablation provided the following results: concordance=0.662 (standard error=0.023); likelihood ratio test= 41.250 on 7 df, p<0.00001; Wald test=36.080 on 7 df, p<0.00001. The assessment of heterogeneity from the 8 curves of the controls provided the following results: concordance=0.631 (standard error=0.02); likelihood ratio test= 32.270 on 7 df, p=0.0004; Wald test=29.760 on 7 df, p=0.0001. Regarding the heterogeneity found in the overall comparative analysis based on the 16 curves, concordance was 0.544 (standard error=0.014), likelihood ratio test was 8.901 on 1 df, Wald test was 8.790 on 1 df, and p-value was 0.003.

Comparison of the results generated by the two methods

In the first place, one should keep in mind that the meta-analysis by Virk and Kumar [6] was based on nine trials, whereas our reanalysis included eight trials. Apart from this difference, the results of the two analyses proved to be very similar (Table 2). Remarkably, the benefit provided by the ablation was found to be more significant in the reanalysis based on eight trials (p=0.003) than in the original analysis based on nine trials (p=0.02).

**Table 2:** Synopsis of the statistical results generated by the two methods. § The HR refers to the comparison of the eight ablation groups vs the eight control groups §§ Three separate heterogeneity assessments (for the analysis of the eight ablation curves, for the analysis of the eight curves of the controls, and for the main comparative analysis evaluating the eight ablation curves vs the eight curves of the controls) gave a statistically significant result HR - hazard ratio; CI - confidence interval

Method	Hazard ratio^§^	95% CI	Significance level	Presence of between-trial heterogeneity
Standard survival statistics	0.820	0.701 to 0.970	p=0.02	No heterogeneity
IPDfromKM method	0.728	0.590 to 0.898	p=0.003	Significant heterogeneity^§§^

Regarding heterogeneity, the graphs reported in Figure 2 and Figure 3 clearly support the presence of a pronounced heterogeneity in this clinical material and, therefore, confirm the validity of the IPDfromKM reanalysis, probably because of its ability to take into account the effect of the follow-up. In contrast, the lack of heterogeneity (I^2^=0%) reported by Virk and Kumar [6] cannot be trusted and likely depends on the use of the RR as opposed to the HR.

## Discussion

Regarding the effectiveness of ablation in preventing ventricular tachycardia, our results confirm those of Santangeli et al. [17] and Virk and Kumar [6] and, therefore, offer sound confirmation on this point. By contrast, our results differ from those reported by Shaheen et al. [18], likely because their study was mainly aimed at comparing two different approaches of ablation (endoepicardial vs endocardial) and also because it included a different selection of clinical studies (11 non-randomized studies and no randomized studies). However, one drawback to our comparison between ablation vs no-ablation is that the treatments given to the eight control groups were not the same [5] since they might consist of either antiarrhythmic drugs or antiarrhythmic drugs plus ICD.

Although our investigation was mainly aimed at comparing two different statistical approaches for analyzing a time-to-event dataset, the results generated by the IPDfromKM method represent original findings because this is the first example in the scientific literature in which ablation for preventing ventricular tachycardia has been investigated through a meta-analysis of "reconstructed" individual patients that takes into account the length of the follow-up. Hence, the clinical value of these results is important because the IPDfromKM method allowed a more thorough interpretation of this dataset. 

The methodological comparison between a standard binary meta-analysis and an analysis based on reconstructed patient-level data (Table 2) raises numerous issues. The main finding from our results is that a clear agreement between the two methods was found in estimating the effectiveness of ablation. On the other hand, important differences emerged in the assessment of heterogeneity. These differences likely depend on the ability of the IPDfromKM method to take into account the effect exerted by the follow-up length on the endpoint and are consistent with the well-known advantages of the hazard ratio over the relative risk. All in all, the assessment of heterogeneity was found to be more accurate if the IPDfromKM method is used.

## Conclusions

Two final comments are worthwhile. First, with reference to these clinical data sets, the IPDfromKM method offers a useful description of the pattern of event occurrence over time, which cannot be obtained through a standard binary meta-analysis; furthermore, a descriptive representation of individual trials is provided, which improves the reporting and interpretation of between-trial variability. Second, the original survival outputs of the IPDfromKM method represent a form of supplementary information directly based on reconstructed patients, which represents an important completion of a standard meta-analysis. Gaining more familiarity with these graphs is worthwhile because the potential advantages of these methodological tools will increasingly be recognized as more and more research is conducted in this area. For example, the visual inspection of the Kaplan-Meier graphs of the individual trials is an efficient tool both to assess between-trial heterogeneity and to estimate the magnitude of the incremental benefit.

In conclusion, the article offers a significant contribution to the literature on meta-analysis methodology and clinical trial data interpretation. The introduction of the IPDfromKM method is particularly promising and warrants further research and applications.
